# How should systematic reviewers handle conference abstracts? A view from the trenches

**DOI:** 10.1186/s13643-019-1188-0

**Published:** 2019-11-07

**Authors:** Roberta W. Scherer, Ian J. Saldanha

**Affiliations:** 1 Center for Clinical Trials and Evidence Synthesis, Department of Epidemiology, Johns Hopkins Bloomberg School of Public Health, 615 N. Wolfe Street, Room E6138, Baltimore, MD 21205 USA; 2 Center for Evidence Synthesis in Health, Department of Health Services, Policy, and Practice (Primary), Brown University School of Public Health, Providence, RI USA; 3 Department of Epidemiology (Joint), Brown University School of Public Health, Providence, RI USA

## Abstract

**Background:**

While identifying and cataloging unpublished studies from conference proceedings is generally recognized as a good practice during systematic reviews, controversy remains whether to include study results that are reported in conference abstracts. Existing guidelines provide conflicting recommendations.

**Main body:**

The main argument for including conference abstracts in systematic reviews is that abstracts with positive results are preferentially published, and published sooner, as full-length articles compared with other abstracts. Arguments against including conference abstracts are that (1) searching for abstracts is resource-intensive, (2) abstracts may not contain adequate information, and (3) the information in abstracts may not be dependable. However, studies comparing conference abstracts and fully published articles of the same study find only minor differences, usually with conference abstracts presenting preliminary results. Other studies that have examined differences in treatment estimates of meta-analyses with and without conference abstracts report changes in precision, but usually not in the treatment effect estimate. However, in some cases, including conference abstracts has made a difference in the estimate of the treatment effect, not just its precision. Instead of arbitrarily deciding to include or exclude conference abstracts in systematic reviews, we suggest that systematic reviewers should consider the availability of evidence informing the review. If available evidence is sparse or conflicting, it may be worthwhile to search for conference abstracts. Further, attempts to contact authors of abstracts or search for protocols or trial registers to supplement the information presented in conference abstracts is prudent. If unique information from conference abstracts is included in a meta-analysis, a sensitivity analysis with and without the unique results should be conducted.

**Conclusions:**

Under given circumstances, it is worthwhile to search for and include results from conference abstracts in systematic reviews.

## Background

Systematic reviewers aim to be comprehensive in summarizing the existing literature addressing specific research questions. This generally involves a thorough search for published studies as well as for ongoing or recently completed studies that are not yet published. Ongoing and recently completed studies are often identified through searches of registries, such as ClinicalTrials.gov, and of conference proceedings. While identifying and cataloging unpublished studies from conference proceedings is generally recognized as a good practice during systematic reviews, controversy remains whether to include study results that are reported in conference abstracts. Current guidelines are conflicting. The United States Agency for Health Care Research and Quality (AHRQ), through its Effective Healthcare Program, recommends that searches for conference abstracts be *considered*, but Cochrane and the United States National Academy of Sciences (NAS) both recommend *always searching for and including* conference abstracts in systematic reviews [1–3]. Our objectives in this commentary are to summarize the existing evidence both for and against the inclusion of conference abstracts in systematic reviews and provide suggestions for systematic reviewers when deciding whether and how to include conference abstracts in systematic reviews.

## Main text

### Arguments for including conference abstracts in systematic reviews

The main argument for including conference abstracts in systematic reviews is that, by doing so, systematic reviewers can be more comprehensive. In our recent Cochrane methodology review, we reported that the proportion of subsequent full publication of studies presented at conferences is low [4]. We examined 425 biomedical research reports that followed the publication status of 307,028 studies presented as conference abstracts addressing a wide range of medical, allied health, and health policy fields. A meta-analysis of these 425 reports indicated that the overall full publication proportion was only 37% (95% confidence interval [CI], 35 to 39%) for abstracts of all types of studies and only 60% (95% CI, 52 to 67%) for abstracts of randomized controlled trials (RCTs). Through a survival analysis, we found that, among the 181 reports that evaluated time to publication, only 46% of abstracts of all types of studies and 69% of abstracts of RCTs were published, even after 10 years. Thus, at best, approximately 3 in 10 abstracts describing RCTs have never been published in full, implying that the voluntary participation and risk-taking by multitudes of patients have not led to fully realized contributions to science. We and others argue that the failure of trialists to honor their commitment to patients (that patient participation would contribute to science) represents an ethical problem [5, 6].

From a systematic reviewer’s perspective, even if the unpublished abstracts were a random 3 in 10 abstracts, restricting a systematic review search to only the published literature would amount to the loss of an immense amount of information and a corresponding loss of precision in meta-analytic estimates of treatment effect. However, publication is not a matter of random chance. Those conducting systematic reviews have long grappled with this problem, known as “publication bias.” Publication bias occurs when either the likelihood of, or the time to, publication of a study is impacted by the direction of the study’s results [6–12]. The most frequent scenario for publication bias is when studies with “positive” (or “significant”) results are selectively published, or are published sooner, than studies with either null or negative results.

Publication bias can be conceptualized as occurring in two stages: (I) from a study’s end to presentation of its results at a conference (and publication of an accompanying conference abstract) and (II) from publication of a conference abstract to subsequent “full publication” of the study results, typically in a peer-reviewed journal article [13]. In the context of publication bias arising during stage II (i.e., if abstracts with positive or significant results are selectively published in full), systematic reviews relying solely on fully published studies can be biased because positive results would be overrepresented. This would lead to a falsely inflated (or biased) estimate of the treatment effect of the intervention being evaluated in the systematic review. Indeed, in our Cochrane methodology review, we found evidence of publication bias in the studies reported in the abstracts [4]. “Positive” results were associated with full publication, whether “positive” was defined as statistically significant results (risk ratio [RR] = 1.31, 95% CI 1.23 to 1.40) or as results whose direction favored the intervention (RR = 1.17, 95% CI 1.07 to 1.28). Furthermore, abstracts with statistically significant results were published in full sooner than abstracts with non-significant results [14–16], unearthing another aspect of bias that can arise when a systematic review is performed relatively soon after the completion of a trial(s) testing a new intervention.

### Arguments against including conference abstracts in systematic reviews

There are various arguments against including abstracts in systematic reviews. First, identifying relevant conferences, locating their abstracts, and sifting through the often thousands of abstracts can be challenging and resource-intensive. However, EMBASE, a commonly searched database during systematic reviews, now includes conference abstracts from important medical conferences, dating back to 2009 [17]. Inclusion of conference abstracts in this searchable database means searching for conference abstracts is less resource-intensive than in the past. Second, largely driven by their brevity, abstracts may not contain adequate information for systematic reviewers to appraise the design, methods, risk of bias, outcomes, and results of studies reported in the abstracts [18–21]. Third, the dependability of results presented in abstracts also is questionable [22–24], which occurs at least in part because (1) most abstracts are not peer-reviewed and (2) results reported in abstracts are often preliminary and/or based on limited analyses conducted in a rush to meet conference deadlines. The most frequent types of conflicting information between abstract and full-length journal article have pertained to authors or authorship order, sample size, and estimates of treatment effects (their magnitude or, less frequently, direction) [25–31]. Mayo-Wilson and colleagues examined the agreement in reported data across a range of unpublished sources related to the same studies in bipolar depression and neuropathic pain [21, 32]. As part of this effort, they compared abstracts with full-length journal articles and clinical study reports and reported that the information presented in abstracts was not dependable either in terms of methods or results.

### What are we missing if we do not include conference abstracts in a systematic review?

Various studies have questioned whether the inclusion of “gray” literature or unpublished study results in a systematic review would change the estimates of treatment effect obtained during meta-analyses. Through “meta-epidemiologic” studies, investigators have examined the results of meta-analyses with and without conference abstracts and have reported conflicting, but generally small differences in results [21, 24, 33]. Evidence from a recent systematic review indicates that the inclusion of gray literature (defined more broadly than just conference abstracts) in meta-analyses may change the results from significant to non-significant or from non-significant to significant, or may not change the results [24, 33]. We conducted a similar analysis in our Cochrane methodology review [4]. We were able to do this because some of our included reports that examined full publication of conference abstracts were themselves only available as conference abstracts. Our analysis found that inclusion of reports that were conference abstracts did not change the strength or precision of our meta-analytic results. In our review, it would have been possible to exclude conference abstracts and retain accurate and precise results.

### Implications of reasons for non-publication of conference abstracts

The most common reason provided by authors of abstracts for not publishing their study results in full has been reported to simply be “lack of time,” and not because the results were considered unreliable or negative [34]. This finding suggests that the identification of an abstract without a corresponding full-length journal article should prompt systematic reviewers to search for additional evidence, such as gray literature sources and/or contacting the authors. However, a reasonable argument could be made that, when the same information is available in both a published peer-reviewed article and an abstract for a given study, including the abstract in a systematic review would be superfluous and/or ill-advised because a likely more comprehensive and dependable source of the information, i.e., the peer-reviewed article, is available. Therefore, the presence of a journal article might obviate the need for including a corresponding conference abstract in a systematic review, unless unique outcomes are reported in the abstract.

### Considerations when including conference abstracts in systematic reviews

Taken together, the evidence reviewed in this paper (summarized in Table 1) suggests that systematic reviewers should take a more nuanced approach to inclusion of conference abstracts. A simple yes or no to the question “Should we include conference abstracts in our systematic review?” is neither sufficient nor appropriate. One aspect to consider is the scope of the review. For example, will the conference abstracts be used to inform policy based on a cadre of systematic reviews or only used within a single review? Benzie and colleagues evaluated the usefulness of including conference abstracts in a “state-of-the-evidence” review and concluded that including conference abstracts validated the results of a search that included only the published literature [35]. These authors discussed four considerations for basing the decision to include conference abstracts: (1) complexity of the intervention, (2) consensus in the existing literature, (3) importance of context in evaluating the effect of the intervention, and (4) presence of other evidence [35]. Others who have incorporated conference abstracts for decision-making have noted that the lack of, or conflicting results in, published evidence often requires inclusion of conference abstracts [36]. In some instances, results in abstracts can confirm the evidence found in fully published studies, but in other instances, abstracts can provide useful additions to the evidence [37].

**Table 1:** Arguments for and against including conference abstracts in systematic reviews

For	Against
Comprehensiveness increased	Resources may be inadequate for locating relevant conference abstracts
Potential for impact of publication bias decreased	Abstracts may not contain adequate information for systematic reviewers to appraise the design, methods, risk of bias, outcomes, and results of studies reported in the abstracts
Increased information leads to increased precision	Dependability of results presented in abstracts is questionable

When considering the use of conference abstracts in systematic reviews, we largely agree with the recommendations presented in the AHRQ Methods Guide for Comparative Effectiveness Reviews [1]. Although these recommendations generally do not espouse including conference abstracts in systematic reviews, they provide excellent guidance on when including abstracts should be considered:

• Reviewers should routinely consider conducting a search of conference abstracts and proceedings to identify unpublished or unidentified studies.

• Consult the TEP [Technical Expert Panel] for suggestions on particular conferences to search and search those conferences specifically.

• Search for conference abstracts of any conference identified by reading the references of key articles.

• We do not recommend using conference abstracts for assessing selective outcome reporting and selective analysis reporting, given the variable evidence of concordance between conference abstracts and their subsequent full-text publications [1].

### Our suggestions

**Fig. 1:**
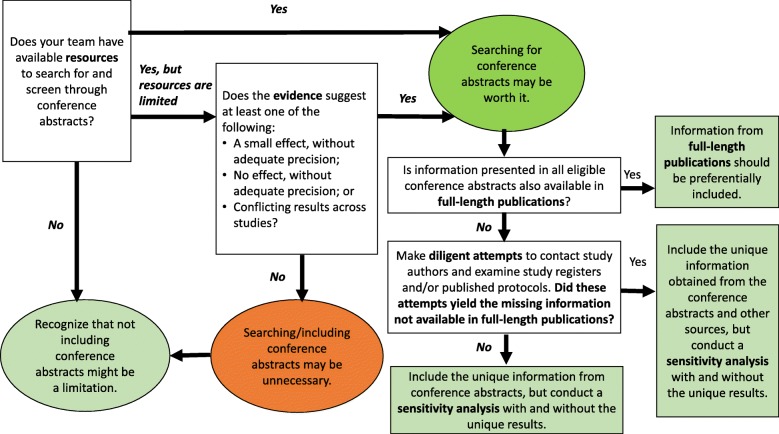
Flow chart showing our suggestions for how to approach the use of conference abstracts in systematic review

Based on the empirical findings summarized in this review and on our experience, we believe that generally relying on conference abstracts is problematic for the various reasons discussed. While meta-epidemiologic studies have shown that inclusion of abstracts does not greatly impact meta-analytic results, it can sometimes make a difference. The dilemma facing a systematic reviewer is to determine when it might. We suggest the following approach (summarized in Fig. 1). If the evidence suggests a sizeable effect, or the absence of one (i.e., with the estimate of effect centered at or near the null), with reasonable precision, searching for conference abstracts may be unnecessary. On the other hand, if the evidence does not show a sizeable effect, is imprecise, or is conflicting, then the resources spent finding and including conference abstracts may be worth it. In other words, if only a single study in full-length form is identified, or if the studies identified are few and small, then conference abstracts should probably be searched and included. We refrain from making specific suggestions for what should be construed as a “sizeable” effect. Magnitudes of effect sizes and thresholds for what is considered relevant can vary considerably across outcomes and across fields and disciplines. We also refrain from making specific suggestions for what should be construed as “reasonable precision” because of the various problems inherent in the use of statistical significance (e.g., arbitrariness, dependence on sample size) and the arbitrary thresholds for precision that use of statistical significance can engender [38–41].

If abstracts are indeed included in a systematic review, the consistent use of CONSORT reporting guidelines for abstracts [14] would facilitate extraction of information from abstracts. In many cases, however, these reporting guidelines are not followed [42], so we suggest that diligent attempts be made to contact authors of the abstracts and examine study registers, such as ClinicalTrials.gov, and published protocols to obtain all necessary unreported or unclear information on study methods and results. In addition, to examine the impact of including the abstracts, a sensitivity analysis should always be completed with and without conference abstracts.

## Conclusions

Based on the available evidence and on our experience, we suggest that instead of arbitrarily deciding to include conference abstracts or not in a systematic review, systematic reviewers should consider the availability of evidence. If available evidence is sparse or conflicting, it may be worthwhile to include conference abstracts. If results from conference abstracts are included, then it is necessary to make diligent attempts to contact the authors of the abstract and examine study registers and published protocols to obtain further and confirmatory information on methods and results.

## Data Availability

Not applicable.

## Abbreviations

AHRQ: Agency for Health Care Research and Quality
CI: Confidence interval
NAS: United States National Academy of Sciences
RCT: Randomized controlled trial
RR: Risk ratio
